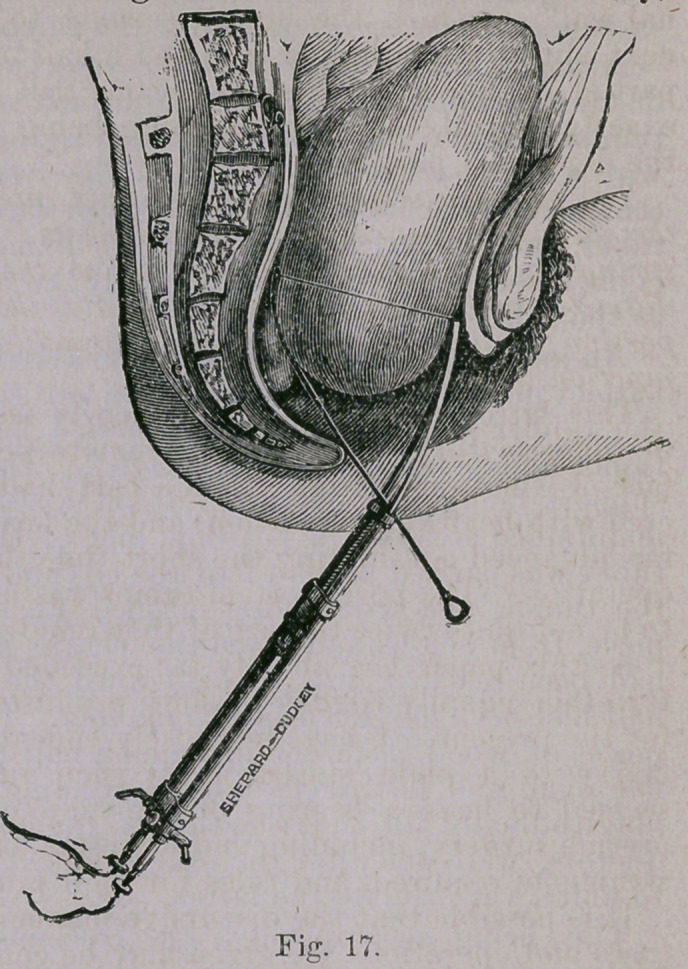# Clinical Notes on the Electric Cautery in Uterine Surgery

**Published:** 1873-03

**Authors:** J. Byrne

**Affiliations:** Surgeon-in-Chief to St. Mary’s Hospital for Diseases of Women; Clinical Professor of Uterine Surgery to Long Island Medical College, etc.


### Miscellaneous.

### Clinical Notes on the Electric Cautery in Uterine Surgery.

BY J. BYRNE, M. D.,

*Surgeon-in-Chief to St. Mary's Hospital for Diseases of Women ; Clinical Professor of Uterine Surgery to Long Island Medical College, etc.*

### CASE XVII.

AMPUTATION OF CERVIX UTERI FOR HYPERTROPHY AND PROCIDENTA, RESULTING IN PERMANENT ELEVATION OF THE UTERUS.

Mrs. ——, aged 35, has had five children, the youngest 3½ years, and one miscarriage about three years previous to my seeing her, which was on December 16th, 1870. Complained of severe and constant backache, bearing-down pains, leucorrhœa and vesical tenesmus. Menstruation regular, though somewhat painful, and occasionally in the intervals more or less muco-sanguineous discharge, especially after long standing or fatiguing exercise. On examination per veginam, the uterus was found low down, immediately within the vulgar outlet, and the cervix much enlarged, irregular in form, and tender. Os tincæ sufficiently open to admit the point of finger, but not further dilatable on account of the swollen condition of surrounding tissues.

The vesical wall was dragged down to such a degree as to constitute cystocele when the patient stood erect. The finger, on being withdrawn, was covered with a sanious mucus. The speculum being now introduced, the appearance of the organ was such as might be expected, the cervix fully two and one-half inches in diameter, purplish-red, and lobulated. The sound passed to the extent of four inches, and in such a direction as to show some degree of anteversion with slight flexion; but by conjoined manipulation it was evident that the great depth of the uterus was due to the increased size of its cervix, and that there was little or no corporal hypertrophy.

After a few months' treatment, consisting principally of warm vaginal douches, ido-glycerine to cervix, a Hodge's pessary, etc., the uterus improved greatly, and she stopped visiting the out-door department of the hospital for some time.

January 4, 1872, she applied again for advice, and stated that her former improvement did not continue long.

Her general physical condition was now much changed for the worse, and she had several attacks of protracted menorrhagia since last seen. The depth of the uterus was four inches, and, except that the most gentle introduction of the sound caused hemorrhage from the cervicle membrane, the parts presented an appearance very similar to that first observed.

She was advised to come into hospital for operation, and did so on Feb. 2, 1872, when it was decided to remove the whole cervix close to its vaginal insertion, by galvano-cautery, and subsequently, when the parts would heal, to take away portions of the anterior vaginal wall by Dr. J. C. Nott's clamp-ecraseur.

*Operation.*—By means of the small cautery-knife (G) a circular fissure was made around the base of the cervix so as to form a bed for the wire-loop. The latter was next adjusted and the part to be removed securely embraced, while *slight* traction was made by means of vulsellum. (See Fig. 10.)

The battery connection being now effected, the loop was *slowly* contracted, so as to occupy not less than eight or ten minutes in passing through, thereby avoiding hemorrhage. When the cervix was lifted out the stump was found to be deeply concave; and as there was no appearance of blood, neither tampon nor other dressing was applied.

During the three days subsequent to the operation, no special treatment was needed, as the patient felt no inconvenience whatever from what had been done.

About the fourth day, which I find is the rule in such cases—a copious discharge of healthy pus began to flow, and during the ensuing week the vagina was douched twice a day with tepid water and castile soap, and at a later period with a solution of sulphate of zinc and water (ʒ i. to Oi.). An examination made on the 2d March (four weeks after operation) showed the parts to be entirely healed, and the surface from which the cervix had been removed, *smooth* and covered with healthy membrane.

March 9th.—The patient was placed upon the table, and anæsthetized previous to operating on the anterior wall, as above stated, my friend Dr. Nott and the members of the hospital staff being present, when, to the surprise of all, the following condition of things was observed: *There was no bulging of the vesico-vaginal septum, and the uterus was with difficulty reached by the finger, as if the vaginal canal had been stretched in an upward direction. The uterus was not alone elevated, but no reasonable amount of traction, by means of a vulsellum, could move it from its lofty position.* No further operations being indicated, she was soon after discharged cured.

This remarkable degree of fixation of the uterus, following amputation of its cervix by the electric cautery, is a clinical fact worth bearing in mind, especially as neither fever, pelvic or adominal pain, nor, in fact, any other symptom indicative of inflammatory action, followed the operation. However, there cannot, I think, be a doubt but that it was due to some local inflammation of a subacute form in the areolar tissue and lymphatics of the broad ligaments, resulting in a tightening or abnormal inelasticity of the uterine supports.*

### CASE XVIII.

### INTRA-PELVIC FIBROID.—THIRD OPERATION ON SAME PATIENT.

The young lady whose case has already been fully given (Case XIII), having entirely recovered from the severe ordeal undergone in August last, and having suffered much of late from vesical tenesmus, occasional retention of urine, and other distressing effects of pelvic impaction, was induced to submit to a third operation on first of the present month (December). This consisted in the removal of all that part of the tumor within the lower pelvis, the presence of which was the cause of all the suffering now complained of, and the excision of which at an earlier period did not seem warrantable on account of her weak condition.

The part now referred to may therefore be considered as the stump from which the large mass was taken on the former occasion. It does not seem to have increased in size during the last three months, though its presence has become more and more painfully felt of late. The upper two-thirds of the pelvic cavity was tightly packed, but the inferior portion towards the vaginal outlet was crowded, principally on account of the globular form of the stump. The latter was perfectly smooth, and presented no appearance of having ever been an open granulating surface or being covered with cicatricial tissue.

In reflecting over the measures suggested to my mind for accomplishing its removal, either of two methods appeared practicable,—to repeat the operation first resorted to, by splitting the mass into two parts, and then looping either half; or to attempt its removal in one piece by a loop thrown around the whole circumference of the tumor.

On account of the great length of time occupied, however, not to speak of the almost insurmountable trouble and difficulties experienced on a former occasion, the first of these plans offered but little attraction; and though it seemed at first impossible to devise any means by which a smooth globular mass might be embraced by a wire noose, I decided to make the effort.

The method practised may be described as follows: A large-

* In procedentia, where amputation of the cervix is called for, would not the introduction of a cylinder speculum after operation, and its retention for eight or ten days, insure a permanent elevation of the uterus, and provide against relapse of other parts?

sized hard rubber crotchet needle, rounded at the end, was heated and slighlly bent so as to accommodate itself to the curve of the sacrum and posterior contour of the tumor.

Fig. 16.

A small hole was drilled transversely near its distal extremity, and at right angles with the direction of its curve, and through which a stout platina wire was passed half its length. The free ends of the wire were now passed through two copper tubes, each 3-16ths of an inch in diameter, and eight inches long, and bent to nearly the same form as the rubber rod (Fig. 16).

An anæsthetic having been administered, and the patient placed on the left side, the two tubes with the rubber rod between were carried behind the tumor and as far up as deemed safe.* The rubber support being now entrusted to an assistant, and maintained steadily in position, one of the copper tubes was carried around half the circumference of the tumor, the wire being pushed up, piece by piece, from below, and when the centre anteriorly had been reached, was so held until the opposite half had been encircled in like manner. Two small pieces of wood, each one inch and a half in length, flat oval, and having two holes running through longitudinally for the reception of the copper conductors, were one after the other slipped up so as to unite, yet insulate the latter.

This being accomplished, the free ends of the platina wire were next passed through a modification of the loop instrument as shown in Fig. 2, and the copper conductors firmly fastened in the socket. All being now in readiness, the battery connections were made, when the heated wire cut through the rubber support and embedded itself in the substance of the tumor.**

The rubber rod was now withdrawn, and the loop *very slowly* contracted, the time occupied in cutting through the whole mass being fully thirty minutes, exclusive of necessary interruptions. There was no hemorrhage from the stump, but the vagina was tamponed as a precautionary measure.

Reaction after the operation was, in this instance also, quite satisfactory; and though her pulse for several days did not get below 110, she expressed

* Fearing that some abnormal position of the Douglas *cul-de-sac* might exist, the part selected for looping was some distance below the fornix vaginæ.

** On account of the length of wire required to encircle the tumor, two batteries were connected and used until a part of the mass was cut through, after which one was found sufficient.

herself as feeling very comfortable and free from abdominal pain or tenderness. The vaginal dressings were removed on the third day, the parts well bathed and with tepid soap and water; to which was added carbolic acid. Copious discharges of healthy pus now appeared, the vagina was douched several times a day, she enjoyed and retained her nourishment and stimulants, and everything progressed favorably up to the night of the 10th, nine and a half days after the operation. On that night the weather suddenly became intensely cold, and being nervously apprehensive that urine might accumulate in the bladder so as to require the use of a catheter, she persisted in getting out of bed a number of times to pass water.

Fig. 17.

At an early hour of the morning of the 11th, Dr. Schapps saw her, was told she had several chills, and recognized well-marked symptoms of incipient tetanus. This condition of things rapidly became worse, and though every means at our command was promptly applied and persevered in, no amelioration of her spasms was effected thereby, and she died at four A. M. on the 14th.

*Autopsy.*—An incision was made from the ensiform cartilage to the symphysis pubis, and the integuments dissected from the latter preparatory to its removal. This being effected, a careful inspection of the abdominal and pelvic contents *in situ* was thus afforded. There was almost a total absence of adhesions, or any evidence of recent or remote peritoneal inflammation. The ovaries were small and shrivelled, but healthy, and the tubes, with their peritoneal attachments, were free and in other respects normal.

The utero-ovarian plexus of veins on right side was in a varicose condition, and one fully as large as the jugular issued from the outer circumference of this varix, and passed directly upward to a point opposite the gall-bladder, where it entered the ascending cava. The fundus uteri was cupshaped, as if partially inverted; the bladder was healthy; and the peritoneal surfaces all over remarkably pale and free from lymph deposits. The anterior vaginal wall, of which the uterus seemed to be a continuation, was next slit up to within an inch and a half of the fundus, when the partial inversion referred to became still more manifest, and was exactly central, each tubal opening being the lateral boundary of the depressed part.

*The tumor was now found to be not interstitial, but connected to the uterus by two separate attachments: one, the pedicle proper, springing from the right wall below the Fallopian opening, in diameter about two inches, and short: the other covering a great portion of the opposite side, and extending down the cervix to its junction with the vagina.**

This latter connection was evidently secondary, and the result of inflammatory action at some remote period. The vaginal surface of the tumor, from which a part had been excised, was covered with healthy granulations and the healing process remarkably far advanced considering the short time that had elapsed since the operation. The post mortem tumor was not weighed, but appears to be not quite twice the size of that removed by the last operation.

As this paper has already far exceeded its proposed limits, and for other equally cogent reasons, my history of cases must close for the present. I have purposely endeavored to confine my remarks to a plain statement of such facts and occurrences as seemed to have a bearing on the value of the electric cautery in uterine surgery, including a description of the apparatus and instruments required, and rules for their practical application.

It is possible that the discursive manner in which my reports of cases and operations are given may be considered too inexact and disjointed; but I would state, in explanation, that this paper is written less with a view to instruct students than for the information it may convey to active members of the profession; so that the dry daily record and minute details of cases, however useful and necessary to the one class, would be neither attractive nor profitable to the other.

Independently of this feature, however, I am fully aware that my clinical report, as a whole, is neither so full, nor by any means so complete, as could be wished; because, in addition to certain diseased conditions and operations therein described, and which in reality constitute but one-fourth of the whole number observed, there are many others of great practical interest that might also be related did time and other circumstances permit.

Prominent among the latter might be mentioned chronic catarrhal, inflammatory, and ulcerated states of the intra-cervical mucous membrane—as a class, the acknowledged approbrium of gynæcological surgery, but yielding readily and in most instances

* This adhesion of the tumor to the left side of the uterus, undoubtedly resulted from the first attempt made at enucleation in September, 18 9.

to one application of the electric cautery.* Nor indeed does recourse to such radical measures for these obstinate ailments demand the use of any anæsthetic; for patients have repeatedly declared that no more suffering attends or follows such treatment than is observed when any active topical application is made. So also in regard to inflamed and granular states of the urethral membrane, always a source of intense suffering to the patient, and, so far as my own experience goes, but rarely even alleviated by the most judicious methods of treatment ordinarily employed. Yet these painful affections also, when not seriously complicated with vesical lesions, have, in several instances lately met with, disappeared no less rapidly by the same proceeding.†

I regret that, on these points, nothing beyond this mere reference to the facts can be ventured at the present time; but an early opportunity may be taken to submit some clinical illustrations of what may be reasonbly hoped for in such cases.

With regard to the value of galvano-cautery as a means of excising epitheliomatous outgrowths from the uterus, I think sufficient clinical material has been presented to demonstrate, beyond all reasonable doubt, its great superiority over every other mode at our command.

My reports also indicate pretty conclusively the boldness and freedom with which we may, by this agent, safely encounter disease, however intimately connected with vital parts, the securety it affords against hemorrhage, and, what appears to me of even more consequence, the very remarkable immunity it would seem almost to guarantee against peritonitis, cellulitis, pyæmia, and other fatal sequelæ of intra-pelvic operations otherwise effected.

As to the curability of cancroid diseases of the uterus by such radical measures as I have adopted and described, or the degree of permanency thereof reasonably to be hoped for, I have but little to add to the remarks already embodied in my reports. The statistics are, perhaps, as yet too limited, and, in most of my cases, the time that has elapsed since operative treatment is insufficient to warrant any very decided opinion one way or other.

It may not be presuming too much to say, however, that, judging from the apparently complete restoration to health in the great majority of patients so treated, though the condition of some was in the highest degree discouraging at the outset, I cannot hesitate to believe firmly that their ultimate history will warrant the most favorable conclusions in this regard. However, should future observation and more mature experience tend to

* In order to make such applications properly, the cervical cavity should be first well dried out by means of compressed sponge or cotton. The cervical cauterizer should then be introduced as far as may be judged proper, and while COLD The battery is next to be immersed, and during cauterization the instrumont should be rolled half round and back, so that the parts may be equally and well brought under its influence.

† A similar proceeding to that advised for cauterization of the cervical canal should be adopted. The bladder must be completely emptied. and the urethra dried by cotton before introducing the instrnment. An anæsthetic is indispensable in these urethral cases dispel these hopes, and though cases now so full of promise should be found hereafter to have relapsed, it would nevertheless be some consolation to reflect that, in addition to having been instrumental in procuring respite from a painful malady, in no single instance had life been jeopardized by efforts made in behalf of these sufferers. Indeed, this latter remark is substantially applicable to some of the most hopeless forms of carcinoma when treated by galvano-cautery, as may be inferred from a perusal of case XII., and which is but one of several instances met with; for, out of thirteen such cases operated upon, ten were beyond all doubt greatly relieved; and though three only were not improved, none were made worse.

The examples of carcinomatous disease of the uterus, either detailed or referred to in this paper, include nearly every variety described or met with, whether as regards their stage of development, the distinctive characters of their primary elements, or the tissues implicated. Hence it is needless to observe that, so far as the manifestly incurable cases were concerned, the parts involved or removed, the amount of relief afforded, and especially the extent to which life seemed thereby prolonged, varied in proportion to circumstances.

As to those of a less grave nature, they too, as may naturally be presumed, were of different forms and degrees of development, and consequently the steps and limits of operations proportionately varied.

Considering, therefore, all the facts observed in thirty operations, their subsequent progress, and inferences naturally deducible therefrom, the conclusion seems obvious that the electric cautery, when properly employed, is attended with less danger, immediate or remote, and promises better results than can be claimed for any other method of surgical treatment yet devised for such ailments.

It would be interesting, and perhaps profitable, to notice some important points touching the distinctive morbid features characteristic of each case or group; but having neither space nor desire to indulge in pathological hair-splitting or the discussion of questions irrelevant to the subject under consideration, what has been already said must suffice for the present, and may be accepted as a resume of my opinions and convictions. Before disposing of this section of my paper, however, and in conformity with its aim and spirit, I would venture to submit, for the guidance of others, the following aphorisms pertinent to the operative management of this class of cases :

1. In all cases of induration, destructive ulceration, and outgrowths of the cervix uteri of a malignant nature, or believed to be so, and therefore warranting excision by galvano-cautery or other means, such operations should never be limited to the apparent line of demarcation between sound and healthy tissue, but must include the whole vaginal cervix at least, and even more if need be. (See Case I.)

2. When the shape of a part to be excised is such that a loop cannot be made to embrace it, a circular furrow for the reception of the wire may first be made by the cautery knife.

3. The wire-loop, knife, or other instrument should never be brought to a white heat when passing through superficial tissues or cellular growths. (See Cases XVI. and XVII.)

4. Traction on the part to be excised should be carefully avoided until the wire has passed well into the submucous structures.

5. The contraction of the loop should in all cases be very slow and gradual, *yet interrupted*, so as to insure a thorough cauterization of each stratum as passed through.

6. Towards the close of such operations, and as the circle of wire becomes small, let the amount of electricity be proportionately lessened.

7. Apply the knife to the spot intended to be cut *before heating;* and, if possible, be always provided with a duplicate of this little instrument.

8. Shun the use of pursulphate of iron as a utero-vaginal styptic dressing, when possible, and, should any such agent be needed, substitute solutions of alum, or acetic acid, dilute or strong, as circumstances may warrant.

The history of a very remarkable case of fibroid tumor has been described at such length, and the three operations undertaken for its removal in part so fully detailed, that but little need be said in addition to what is contained in the reports.

If, up to this time, proof has been wanting to convince the skeptical, and all who, on purely theoretical grounds, denounce certain forms of galvanic apparatus, because, as they say, their action is not sufficiently constant, these three operations amply furnish it. Others, too, who may have imagined, heretofore, that the galvanic cautery in surgical practice must necessarily be limited to small epitheliomatous or pedunculated tumors, fistulous openings, and birth-marks, will find for the first time how much wider its range of applicability may be extended.

That a highly vascular mass, fifteen inches in circumference, and situated within the pelvic cavity, has been successfully cut through and removed without loss of blood or subsequent inflammatory complications, is a circumstance in the history of galvano-cautery as suggestive as it is worthy of record.

The unfortunate occurrence that brought about a fatal issue in this case after the third operation, namely, exposure to cold, however deeply to be regretted, has nothing whatever to do with the merits of the operation, because up to the time of this accidental misfortune the patient was in a much better condition, and promised a more rapid recovery than at a like period after either of the two previous operations.

The report of an operation for the removal of an intra-uterine sessile fibroid (Case XIV.), exemplifies another and I believe a safer means than that of enucleation, by which the removal of these tumors may sometimes be effected.

Avulsion or enucleation of intra-uterine fibroids is admittedly a hazardous, and at best a most difficult undertaking, because, though encouraging results have occasionally attended the efforts of some surgeons in this direction, the operation is one from which those who are best qualified to appreciate its dangers and difficulties will be most apt to shrink.

I am not aware that any successful attempt has been heretofore made to sever the connection of such an intra-uterine growth as that described in my case, by means of the electric cautery; and though the proceedings therein adopted may be found impracticable in some instances, a persevering effort, when it is deemed possible, would, I think, in a conservative sense, be proper and advisable.

The interest that attaches to the case of fibrous polypus springing from the fundus uteri (Case XV.) is due more to the diagnostic lesson it conveys than to the means by which its removal was effected; because an error in diagnosis, regarding its real point of departure from the uterus, would in all probability have been fatal to the patient. When this tumor was exhibited at a meeting of the New York Obstetrical Society, two examples of this fatal error, in cases precisely similar, were related—one as having occurred in the clinic of Professor Scanzoni within the last two years, and the other in the practice of a prominent New York surgeon. In both cases the fundus uteri, being mistaken for the base of the pedicle, was exterpated, and the patient died in consequence.

Dr. Graily Hewitt,* referring to this subject, says: "When the polypus has a large basis of attachment, the fundus may be so drawn downwards that what appears to us to be the pedicle of the polypus is really the uterus itself. A specimen was not long ago exhibited at the Pathological Society, and referred to Dr. Marion Sims, Dr. John Ogle, and myself for examination, in which such a tumor had been excised, and a circular piece, comprising the fundus uteri, had been removed with it."

I have thought proper, also, to introduce another example of polypus (Case XVI.), the clinical features of which are no less peculiar and instructive than that last referred to. However, as certain inferences deducible from what was noticed in this case have been suggested elsewhere, and important principles, applicable to galvano-cautery, based on facts then observed, have been defined in aphorisms 3 and 4, no further remarks seem called for on the subject.

---

* Diseases of Women, first American from second London Edition, page 529.

Casa XVII. presents some interesting points for reflection, a few of which have already been glanced at in the report. I think this, as well as other similar cases met with, go far towards establishing a fact in the clinical history of such ailments, as well as certain principles applicable to their management, of great practical value.

Thus, however successful Dr. James Henry Bennett and others who accept his pathology and therapeutics of inflammatory and congestive uterine diseases, may have been in "melting down" voluminous cervices by potassa cum calce and other corrosive substances, the most thorough, and by no means superficial, destruction of such parts by the electric cautery, and subsequent copious purulent discharges, cannot be relied on as a remedy for nutritive hypertrophy of the cervix uteri. Moreover, I feel justified in concluding, from my own observation, that amputation of the cervix by galvano-cautery, as compared with local depletion, caustics, and escharotics, offers the quickest, safest, most painless, and by far the most successful treatment for this very numerous class of cases. Whether the explanation already given in regard to the elevated position and immobility of the uterus noticed in this case, is the correct one, or likely to aid us in establishing some principle for our future guidance, will, of course, depend on further experience and the opinion of others.

This much, however, I may add: the circumstance, though probably noticed by others before, appeared so novel to me that I could not well avoid recording it, and the explanation and inferences are offered for what they may be deemed worth.*

In concluding this brief summary of my clinical experience in galvano-cautery I would simply remark that those who confine their appreciation of this invaluable agent in uterine surgery to its blood-saving properties, omit to take into consideration its most attractive and important attributes. These consist, first of all, in the peculiar manner in which this hæmostatic effect is produced on the vessels, and which I surmise is in no way analogous to that effected by ligature, torsion, ecrasement, or styptics. Secondly, as there is no disorganized blood-clots or other effete material to become absorbed into the circulation, blood-poisonings, as I have before observed, need not be apprehended as a sequel of cautery observations.

In other words, it would appear that not only are the blood vessels securely sealed up, but the lymphatics as well, and hence the immunity from hæmatoxic and inflammatory complications.—*Medical Record.*

---

* There is a patient at present under treatment in St Mary's Hospital for vesical and uterine prolapse, and whose future condition will serve to throw some light on these interesting points.

## Figures and Tables

**Fig. 16. f1:**
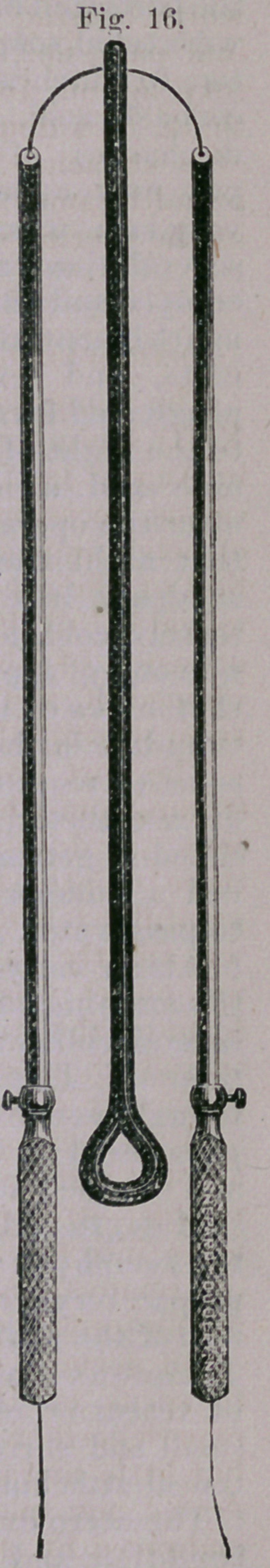


**Fig. 17. f2:**